# Ophiopogonin D Alleviates Nonalcoholic Steatohepatitis via Ferroptosis Through AKT1/STAT3/HIF‐1α Axis

**DOI:** 10.1155/ancp/5567109

**Published:** 2026-03-23

**Authors:** Dong Li, Meina Shi, Zhike Fang, Xiaoqing Wang, Linshi Huang, Mingguo Dong

**Affiliations:** ^1^ Institute of Traditional Chinese Medicine, Dongguan TCM Hospital, Dongguan, 523005, China, itmonline.org; ^2^ Faculty of Chinese Medicine, Macau University of Science and Technology, Macau, 999078, China, must.edu.mo; ^3^ Department of Science and Education, Dongguan TCM Hospital, Dongguan, 523005, China; ^4^ Section XIII Internal Medicine (General Section), Dongguan TCM Hospital, Dongguan, 523005, China

**Keywords:** AKT1, ferroptosis, liver disease, ophiopogonin D, STAT3

## Abstract

**Background:**

Not enough is known about how ophiopogonin D (OP‐D) works and the molecular mechanisms involved in nonalcoholic steatohepatitis (NASH). This study aimed to investigate the antifibrosis effect and underlying mechanism of OP‐D in NAFLD.

**Methods:**

The rats were fed a high‐fat diet (HFD) to simulate NAFLD. After OP‐D treatment or not, the rat serum lipid levels and inflammatory and ferroptosis‐related factors were detected. Online databases and network pharmacology were used to collect the targets of OP‐D against NASH. The effect of OP‐D on AKT1, STAT3, ferroptotic markers, and fibrotic markers was determined using western blot or PCR assays.

**Results:**

OP‐D inhibited abnormal liver function, fibrotic markers (α‐SMA/Col1α1), and ferroptosis‐related factor (GPX4) in NASH rats. Network pharmacology proposed AKT1 and STAT3 as targets for OP‐D against NASH. OP‐D reduced the p‐AKT and p‐STAT3 in liver tissues. OP‐D reduced the levels of AKT1, STAT3, and HIF‐1α in model hepatic stellate cells. AKT1 and STAT3 overexpression reversed the antifibrosis and ferroptosis‐induced effects of OP‐D in model hepatic stellate cells.

**Conclusion:**

OP‐D may alleviate fibrosis in NASH by regulating ferroptosis via the AKT1/STAT3/HIF‐1α axis.

## 1. Introduction

The initial manifestation of nonalcoholic steatohepatitis (NASH) is steatosis within the liver. Dysfunction of lipid metabolism in hepatocytes caused by genetic factors, diet, and insulin resistance will lead to lipid accumulation [1]. The lipotoxicity, oxidative stress, and mitochondrial dysfunction caused by lipid accumulation will further lead to hepatocellular injury and even fibrosis [2]. NASH with liver fibrosis can progress to cirrhosis and even liver cancer, with eventual liver failure or liver transplantation [3, 4]. NASH is still on the rise around the world [5, 6]. It is worth noting that China has the youngest median age and the highest growth rate compared to other countries [7]. In the coming decades, the burden of liver disease in China is predicted to increase substantially. There are currently no effective pharmacologic treatment strategies for NASH, despite its high clinical and socioeconomic burden [8]. NASH treatment is multifaceted and includes known conservative and surgical therapies. Treatment of NASH consists of multimodal interventions, including weight loss, lifestyle changes, and optimizing medications [9]. However, options in pharmacologic treatment for NASH remain limited [10].

The potential for multitarget therapeutics exists in herbs and their active ingredients [11]. For the treatment of NASH, herbal medicine has shown great effects in improving steatosis and inflammation [11]. The multiple mechanisms underlying lipid metabolism and inflammation were found to be involved in the effects of plant medicinal ingredients. Dihydromyricetin repairs liver damage, which may be done by reducing pyroptosis or enhancing ethanol metabolism [12, 13]. Ophiopogonin D (OP‐D) is a C29 steroidal glycoside extracted from the root tuber of *Ophiopogon japonicus*. So far, OP‐D has been found to have a wide range of pharmacological effects, ranging from the treatment of metabolic and immune diseases to cancer treatment [14]. It is also known for its anti‐inflammatory and antioxidant effects in high‐fat diet (HFD)‐induced NAFLD mice [15]. However, reports on the anti‐NASH effects of OP‐D, especially the molecular mechanisms, are insufficient.

Thus, we investigated OP‐D as a new anti‐NASH agent for liver cells, also in an attempt to dissect the mechanism of OP‐D. The effect of OP‐D on ferroptosis has been explored in primary hepatocytes and HepG2 cells.

## 2. Materials and Methods

### 2.1. Reagents

OP‐D (HPLC≥98%) was purchased from MedChemExpress (USA).

### 2.2. Animals and Grouping

Sprague Dawley rats (male, 160–200 g) were from Boehringer Ingelheim (Permission Number SCXK 2022‐0002). The animal experiments were carried out under the supervision of the Animal Research Ethics of Dongguan TCM Hospital. The rats were divided into sham‐operated, HFD, HFD + low‐concentration OP‐D (HFD + L‐OP‐D), HFD + medium‐concentration OP‐D, and HFD + high‐concentration OP‐D, with six rats in each group. Rats in the sham‐operated group were fed standard laboratory food; rats in the HFD group were fed a high‐cholesterol diet, as a standard laboratory diet containing 2% cholesterol, 10% lard, and 2.5% vegetable oil [16]. The total feeding time was 12 weeks under a 12 h light–dark cycle (23 ± 2°C). Treatment groups of rats (HFD + L‐OP‐D, HFD + M‐OP‐D, and HFD + M‐OP‐D groups) were treated with 5, 10, and 15 mg/kg/day of OP‐D by gavage for 7 days, respectively. After treatment, the body weights of the rats were recorded. After euthanasia under carbon dioxide, the rat livers were removed and weighed to obtain the total wet weight of the liver. Rats’ serum and liver tissues were collected and stored at −80°C.

All animal experimental protocols were approved by, and all experiments were performed according to the guidelines of the Animal Ethics Committee of Dongguan TCM Hospital. This study was carried out in compliance with the ARRIVE (Animal Research Reporting In Vivo Experiment) guidelines.

### 2.3. Masson’s Trichrome Staining

The liver tissues were fixed with 4% paraformaldehyde for 48 h, and 4‐μm‐thick paraffin sections were prepared. These sections were stained with Masson trichrome to assess collagen deposition and then photographed at a magnification.

### 2.4. Lipid Detection

The levels of biochemical markers ALT, AST, and TC in rat serum or liver were measured by ELISA kits to evaluate the rat liver function and lipid deposition. The levels of TNF‐α, IL‐6, IL‐1β, and IL‐18 in rat serum were assessed using commercially available kits (Cusabio, China).

### 2.5. Prediction of Targets for OP‐D Against NASH

The potential target identification for OP‐D was conducted using SwissTargetPrediction (http://www.swisstargetprediction.ch/) and PharmMapper Server (https://lilab-ecust.cn/pharmmapper/), access date Feb 11, 2024. NASH‐associated human genes were available from the DisGeNET platform (https://www.disgenet.org/home/) and GeneCards databases (https://www.genecards.org/). The genes obtained from GeneCards were included using “Relevance score” ≥10 as filter criteria. The targets for OP‐D against NASH were obtained by overlapping cohorts of NASH‐associated human genes and OP‐D targets using the Venn online tool. Then, STRING Version 12.0 and Cytoscape software were used as the tools for protein–protein interaction (PPI) analysis. The enriched pathways were predicted using the Oebiotech cloud platform.

### 2.6. Hepatic Stellate Cell Isolation and Culture

Hepatic stellate cells were isolated from HFD model rats as previously described [17]. The obtained cells were suspended in Dulbecco’s modified Eagle’s medium containing 10% fetal bovine serum. The medium was changed every 24 h. Follow‐up experiments were performed using 3–6 generations of semifused hepatic stellate cells. Hepatic stellate cells were treated with 10 μM OP‐D or DMSO.

### 2.7. Western Blotting

After lysis, protein samples were collected from cells or liver tissue. The protein concentration of each protein sample was then determined using the BCA Protein Assay Kit (Beyotime, China). The procedure of the western blot method proceeded by first separating the individual proteins using polyacrylamide gel electrophoresis and then transferring them to a nitrocellulose film solid‐phase carrier to adsorb the proteins. The proteins on the nitrocellulose film were then used as antigens, incubated with the corresponding antibodies, and reacted with horseradish peroxidase (HRP)‐labeled secondary antibodies. After substrate color development, the protein was detected.

### 2.8. Analysis of mRNA Expression

Cells were subjected to total RNA isolation using an RNeasy Mini Kit (Qiagen, Hilden, Germany). RNA was then reverse transcribed using the PrimeScript RT Reagent Kit (Takara Bio, Japan). PowerTrack SYBR Green Master Mix (Thermo Fisher Scientific, USA) was used for quantitative reverse transcription‐polymerase chain reaction analysis on a StepOnePlus Real‐Time PCR System (Thermo Fisher Scientific, USA), with glyceraldehyde‐3‐phosphate dehydrogenase (GAPDH) as the internal standard. The 2^−ΔΔCT^ method was used to calculate relative mRNA expression.

### 2.9. Iron and Malondialdehyde (MDA) Assays

HSCs were cultured and subjected to drug treatments as outlined above. Subsequently, cells were gently harvested in phosphate‐buffered saline (PBS) using a cell scraper. Membrane permeabilization was achieved with 1% Triton, and the resulting lysates were collected via centrifugation. The relative levels of iron and MDA in the lysates were quantified using an Iron Assay Kit (#A039‐2‐1, Jiancheng, China) and an MDA Detection Kit (#A003‐4‐1, Jiancheng, China), strictly following the manufacturer’s protocols.

### 2.10. Statistical Analysis

All experiments were executed with more than three replicates. Two‐group comparisons were made by *t*‐test, and multiple‐group comparisons by one‐way or two‐way analysis of variance analyses. *p*  < 0.05 means significant.

## 3. Results

### 3.1. OP‐D Alleviated Dyslipidemia Induced by HFD in Rats

Masson’s trichrome staining showed significantly higher collagen fiber deposition in the HFD group compared to the normal chow control group, whereas the deposition of collagen fibers was reduced in the OP‐D treatment groups, especially in the high‐dose OP‐D (Figure 1A). Our data analysis showed that the effect of OP‐D on the ratio of liver wet weight to body weight was more intense at a high concentration (Figure 1B). Moreover, the HFD elevated serum ALT and AST, while supplementation with OP‐D reduced them in the HFD‐fed rat (Figure 1C,D). Furthermore, the HFD increased serum TC and TG, as well as hepatic TC and TG, whereas OP‐D effectively reversed these parameters in the HFD‐treated group (Figure 1E–H). These results highlight the alleviation of OP‐D in HFD‐induced weight gain and hyperlipidemia.

Figure 1Ophiopogonin D (OP‐D) had a protective effect against high‐fat diet (HFD)‐induced nonalcoholic steatohepatitis in rats. (A) Representative liver histology visualized by Masson’s trichrome staining. (B) The liver index was expressed as the ratio of the wet weight of the liver to the body weight. (C,D) OP‐D can decrease the rat serum alanine aminotransferase (ALT) and aspartate aminotransferase (AST) levels. (E,F) Total cholesterol (TC) and triglyceride (TG) levels in rat serum. (G,H) Hepatic TC and TG in rats.  ^∗^ *p* < 0.05,  ^∗∗^ *p* < 0.01,  ^∗∗∗^ *p* < 0.001.

**Figure:**
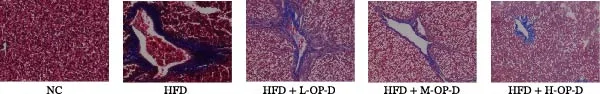
(A)

**Figure:**
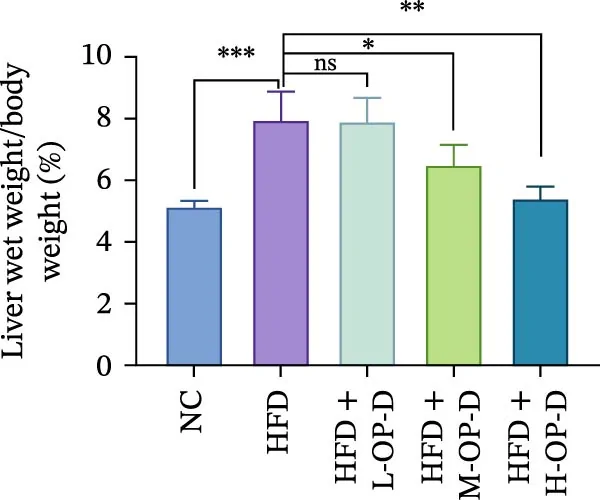
(B)

**Figure:**
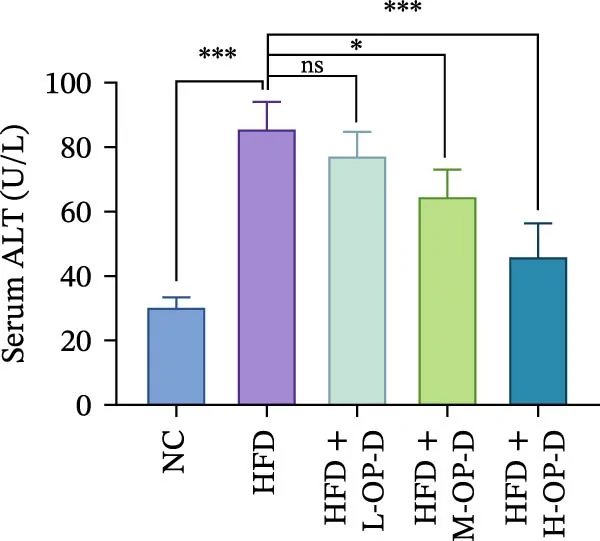
(C)

**Figure:**
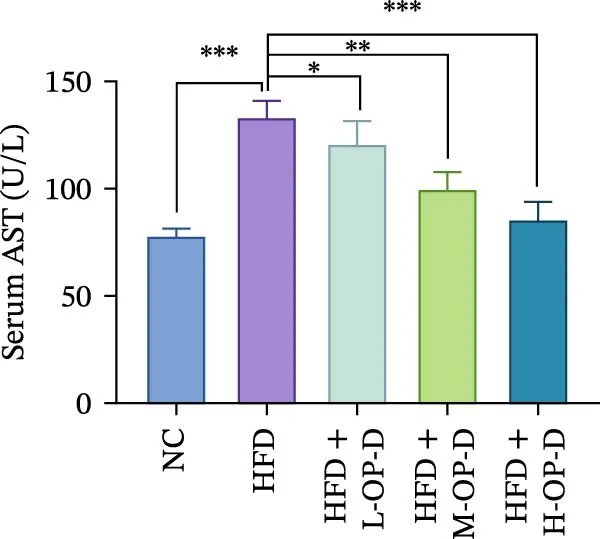
(D)

**Figure:**
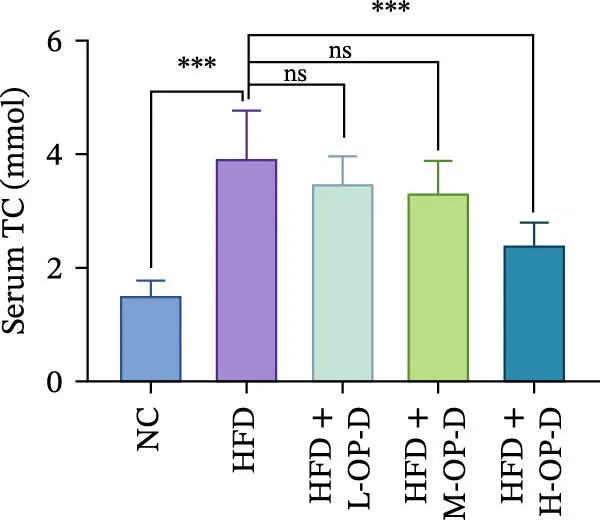
(E)

**Figure:**
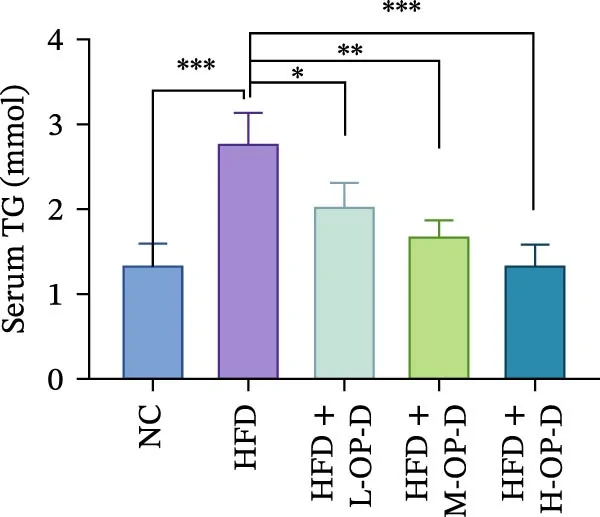
(F)

**Figure:**
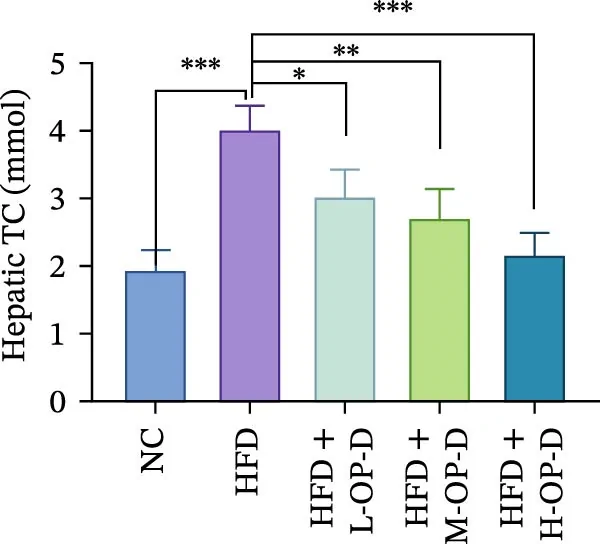
(G)

**Figure:**
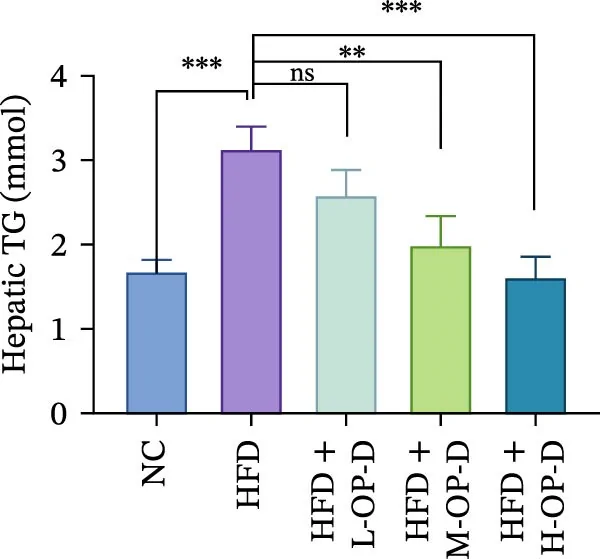
(H)

### 3.2. OP‐D Alleviated Inflammation Induced by HFD in Rats

The changes in the inflammation factor in rat serum are shown in Figure 2. HFD‐fed rats showed marked increases in serum TNF‐α, IL‐6, IL‐1β, and IL‐18 (Figure 2A–D), while supplementation with OP‐D reduced these increases compared with HFD rats (Figure 2A–D). These data indicated that OP‐D can reduce the increase in the secretion of inflammatory cytokines and thereby alleviate liver inflammation during NASH.

Figure 2OP‐D can reduce the rat serum TNF‐α (A), IL‐18 (B), IL‐1β (C), and IL‐6 (D) levels induced by HFD.  ^∗∗∗^ *p* < 0.001.

**Figure:**
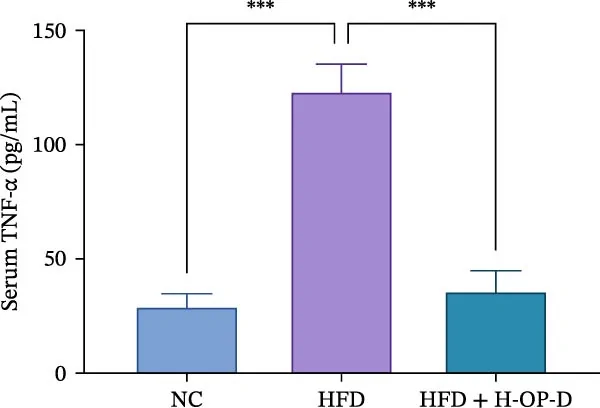
(A)

**Figure:**
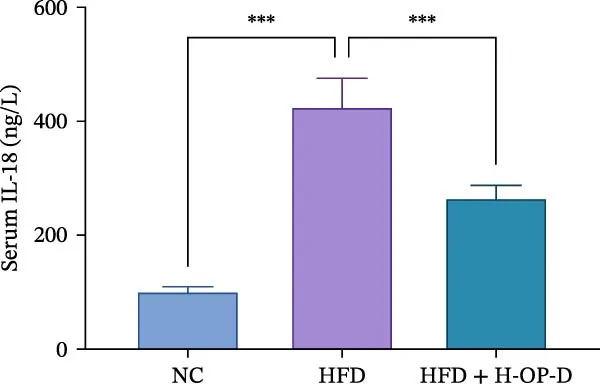
(B)

**Figure:**
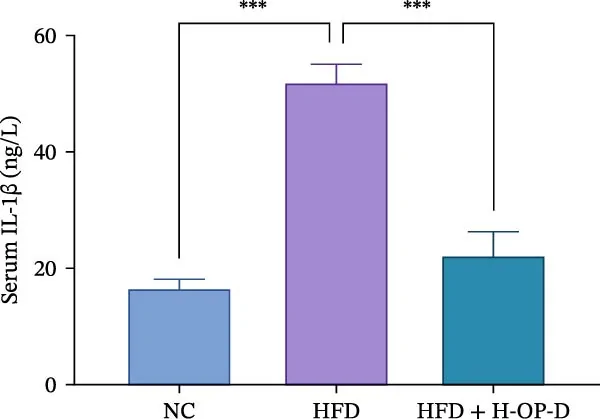
(C)

**Figure:**
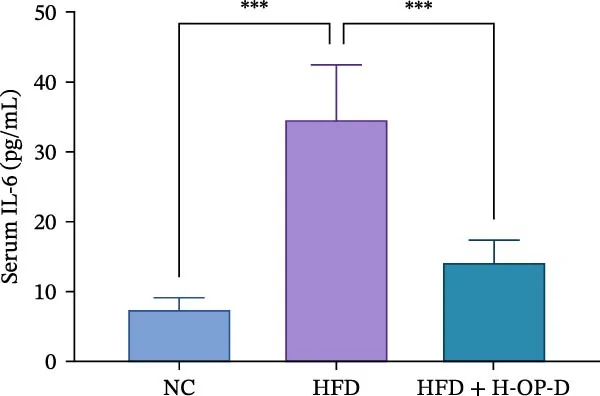
(D)

### 3.3. OP‐D Protected NASH via Multiple Targets

We then use network pharmacology to explore the potential target of OP‐D in NASH. Fourteen targets were overlapped by OP‐D targets and NASH‐related targets from DisGeNET and GeneCards (Figure 3A). The PPIs among these 14 targets proposed AKT1, MMP9, and STAT3 as the top three in degree level (Figure 3B). These genes were enriched in various KEGG pathways, including the HIF‐1 pathway (Figure 3C). Interestingly, AKT1 and STAT3 are involved in the HIF‐1 pathway [18]. Therefore, AKT1 and STAT3 were further investigated.

Figure 3Prediction of targets for ophiopogonin D (OP‐D) using network pharmacology method. (A) The Venn diagram of genes between OP‐D targets and NASH‐related genes from DisGeNET and GeneCards databases. (B) The protein–protein interaction of the proteins by STRING. (C) The KEGG pathway enrichment of the genes.

**Figure:**
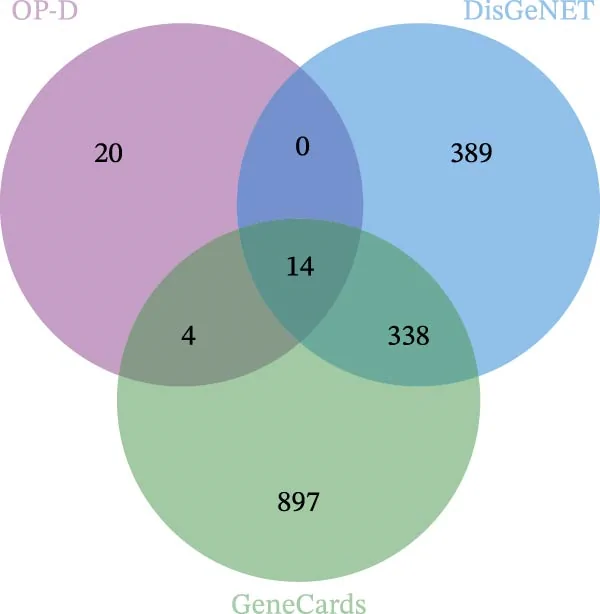
(A)

**Figure:**
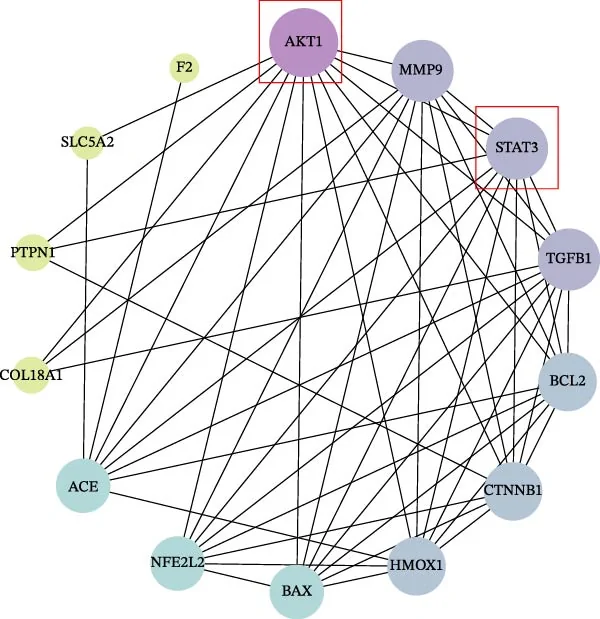
(B)

**Figure:**
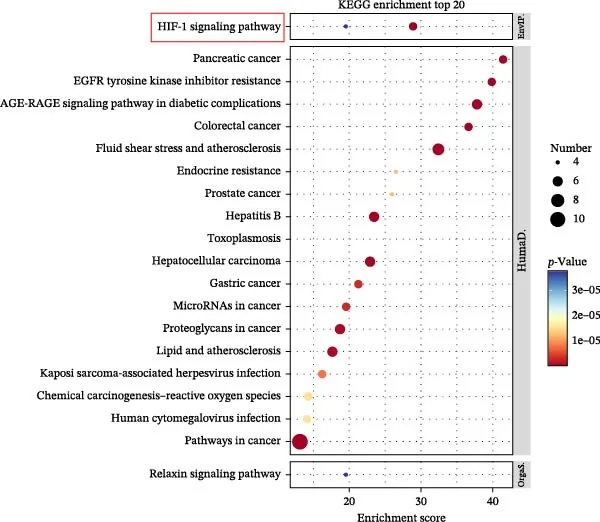
(C)

### 3.4. OP‐D Reduced the Ferroptosis Induced by a HFD in Rats

It is well known that GPX4 and PTGS2 are important regulators in ferroptosis [19–21]. Here, the expression levels of these proteins were detected to explore the sensitivity of ferroptosis in liver tissues with or without treatment with OP‐D (Figure 4A). The expression of GPX4 in rat liver was decreased in the OP‐D‐treated group compared with that in the HFD model group (*p* < 0.01, Figure 4B). The positive monitors of ferroptosis, PTGS2 (*p* < 0.001, Figure 4C), were significantly upregulated in the OP‐D‐treated group, compared with the HFD group. In addition, OP‐D treatment significantly reduced levels of fibrotic signaling factors α‐SMA (*p* < 0.01, Figure 4D) and Col1α1 (*p* < 0.001, Figure 4E). In the rat liver tissues, proteins of p‐AKT1 and p‐STAT3 were determined using western blotting (Figure 4A). OP‐D reduced the levels of AKT1 (*p* < 0.05, Figure 4F) and STAT3 phosphorylation (*p* < 0.05, Figure 4G). Together, these results suggest that ferroptosis was involved in the antifibrosis effect of OP‐D during NASH.

Figure 4Ophiopogonin D (OP‐D) reduced ferroptosis and fibrosis in rat liver. (A) Representative images of western blot protein bands. The ratio of the integrated density values in the western blot bands was quantified and plotted between GPX4 (B), PTGS2 (C), α‐SMA (D), Col1α1 (E), p‐AKT1 (F), and p‐STAT3 (G) to GAPDH in rat livers.  ^∗^ *p* < 0.05,  ^∗∗^ *p* < 0.01,  ^∗∗∗^ *p* < 0.001.

**Figure:**
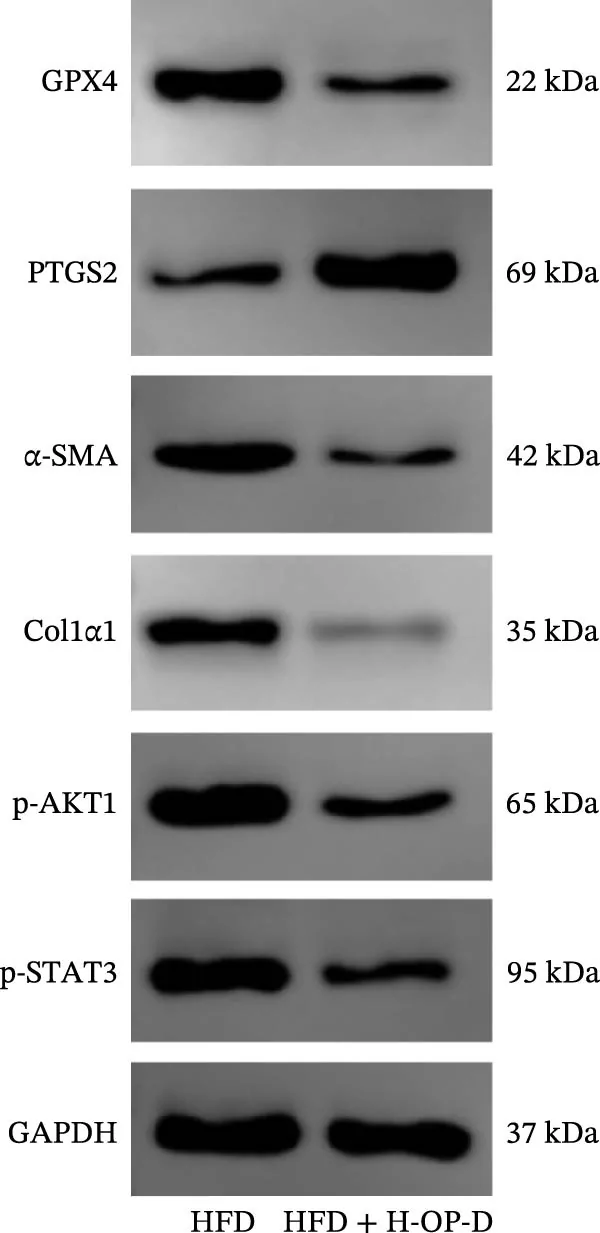
(A)

**Figure:**
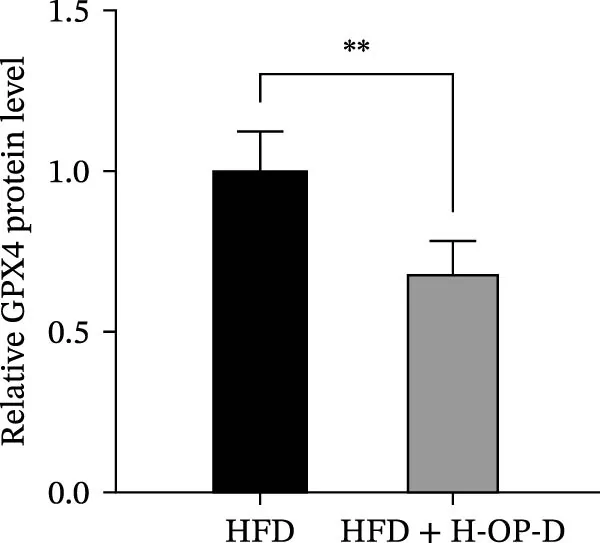
(B)

**Figure:**
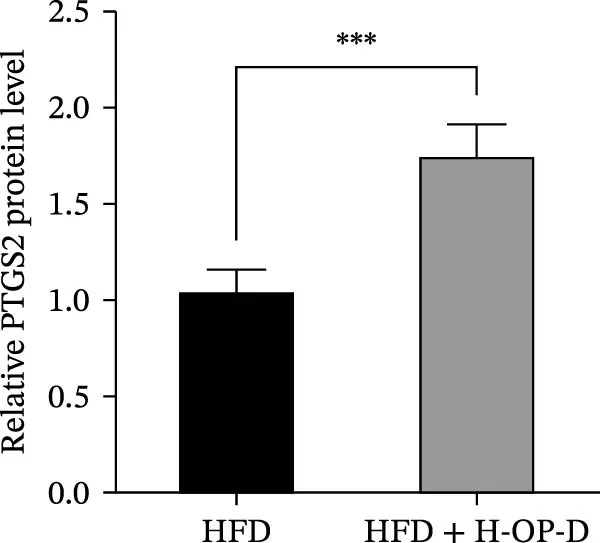
(C)

**Figure:**
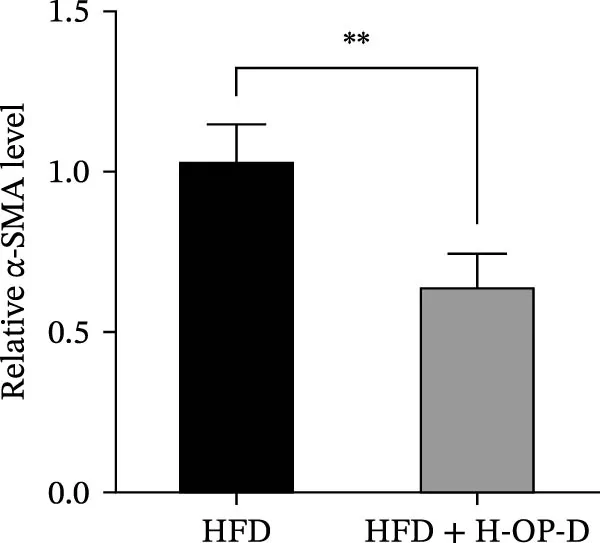
(D)

**Figure:**
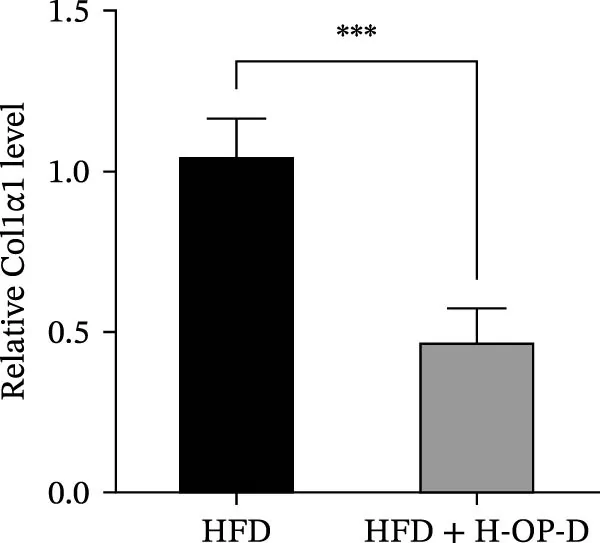
(E)

**Figure:**
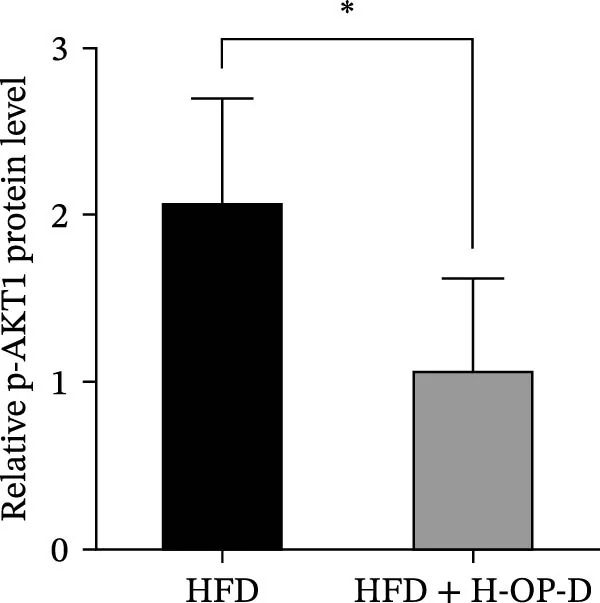
(F)

**Figure:**
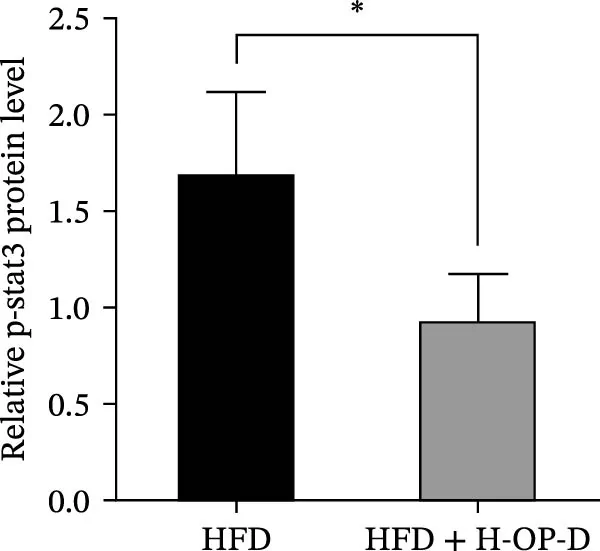
(G)

### 3.5. OP‐D Suppressed AKT1, STAT3, and HIF‐1α in HSC‐T6 Cells

Then, we used OP‐D‐treated HSC‐T6 cells to detect the mRNA expression levels of AKT1 and STAT3. The results showed that after treatment of OP‐D, AKT1 and STAT3 mRNAs were reduced (*p* < 0.01, Figure 5A,B). To further explore the role of the HIF‐1 pathway in the antifibrosis effect of OP‐D, we determined HIF‐1α protein levels in the nucleus and cytoplasm of HSC‐T6 cells. As illustrated in Figure 5C,D, HIF‐1α was decreased in the nucleus but increased in the cytoplasm when treated with OP‐D (*p* < 0.01).

Figure 5Ophiopogonin D (OP‐D) regulated AKT1, STAT3, and HIF‐1α in primary model hepatic stellate cells (HSC). The levels of AKT1 mRNA (A) and STAT3 mRNA (B) in HSC were assayed using RT‐qPCR. HIF‐1α expression in HSC cytoplasm (C) and nucleus (D) was measured by western blot.  ^∗∗^ *p* < 0.01,  ^∗∗∗^ *p* < 0.001.

**Figure:**
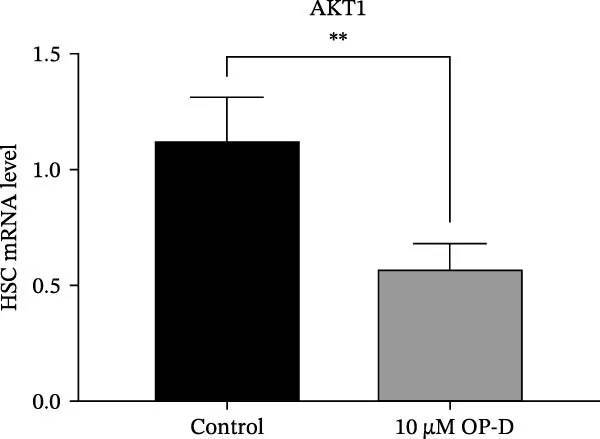
(A)

**Figure:**
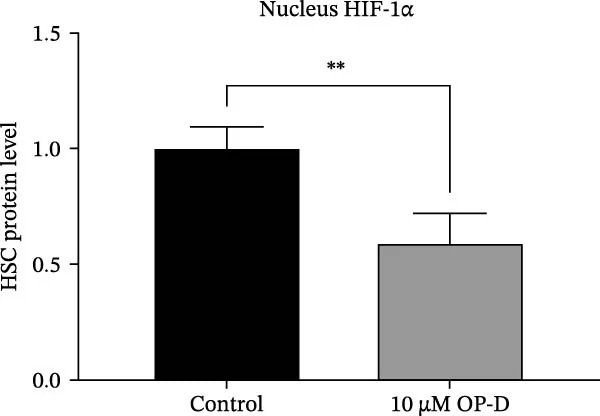
(B)

**Figure:**
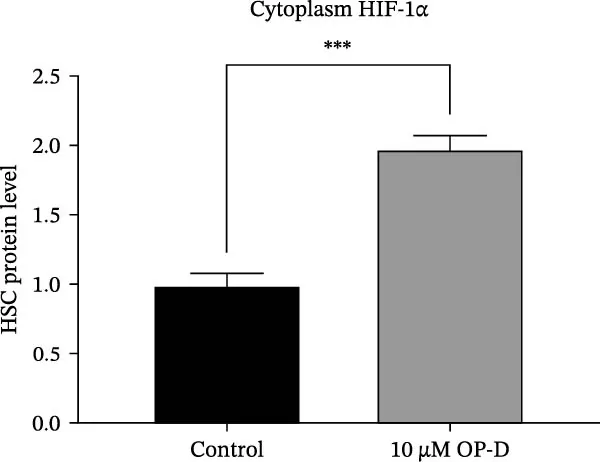
(C)

**Figure:**
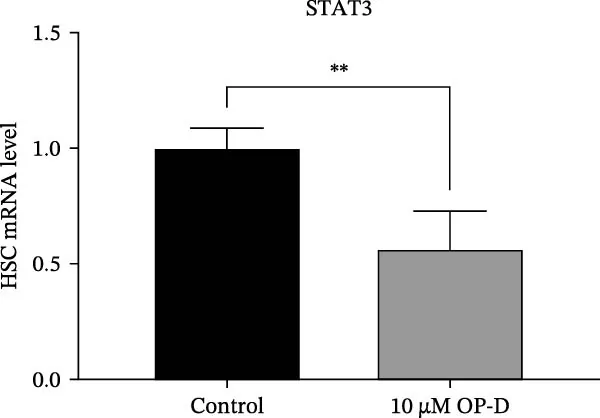
(D)

### 3.6. OP‐D May Confront Liver Fibrosis via AKT1/STAT3/HIF‐1α Axis

To ascertain the involvement of the AKT1/STAT3/HIF‐1α axis in the hepatoprotective effect of OP‐D, pcDNA AKT1/STAT3 was used to transfect HSC‐T6 cells. After transfection, levels of AKT1 and STAT3 mRNA were significantly elevated (*p* < 0.001, Figure 6A,B). Concomitantly, the GPX4 mRNA level was increased (*p* < 0.01, Figure 6C) upon the overexpression of AKT1/STAT3 and the ferroptosis inhibitor ferrostatin‐1. Suppressed ferroptotic events were further evidenced by decreased iron level and MDA content (*p* < 0.01, Figure 6D,E). The protein levels of pathway signals were determined using western blotting (Figure 6F). The effects of OP‐D on protein levels of HIF‐1α (*p* < 0.01) were reversed by the overexpression of AKT1/STAT3 (*p* < 0.01, Figure 6G). The decrease of fibrotic signaling factors α‐SMA and Col1α1 caused by OP‐D treatment was offset by the overexpression of AKT1/STAT3 (*p* < 0.01, Figure 6H,I). These results demonstrate that OP‐D may exert antifibrosis effects by regulating ferroptosis through the AKT1/STAT3/HIF‐1α axis.

Figure 6Ophiopogonin D (OP‐D) regulate hepatic stellate cells (HSC) ferroptosis and fibrosis via AKT1/STAT3. RT‐qPCR assays were conducted for mRNA levels of AKT1 (A), STAT3 (B), and GPX4 (C). (D,E) MDA content and iron release in rat were detected by kits. (F) Representative images of western blot protein bands. The ratio of the integrated density values in the western blot bands was quantified and plotted between HIF‐1α (G), α‐SMA (H), and Col1α1 (I) to GAPDH in HSC.  ^∗∗^ *p* < 0.01,  ^∗∗∗^ *p*  < 0.001.

**Figure:**
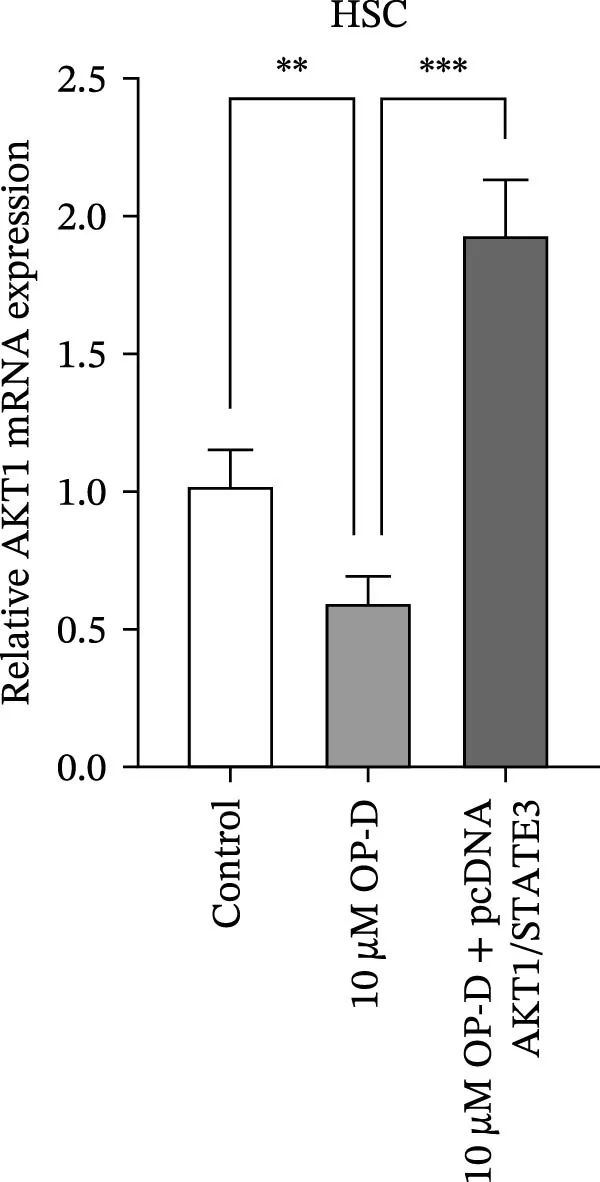
(A)

**Figure:**
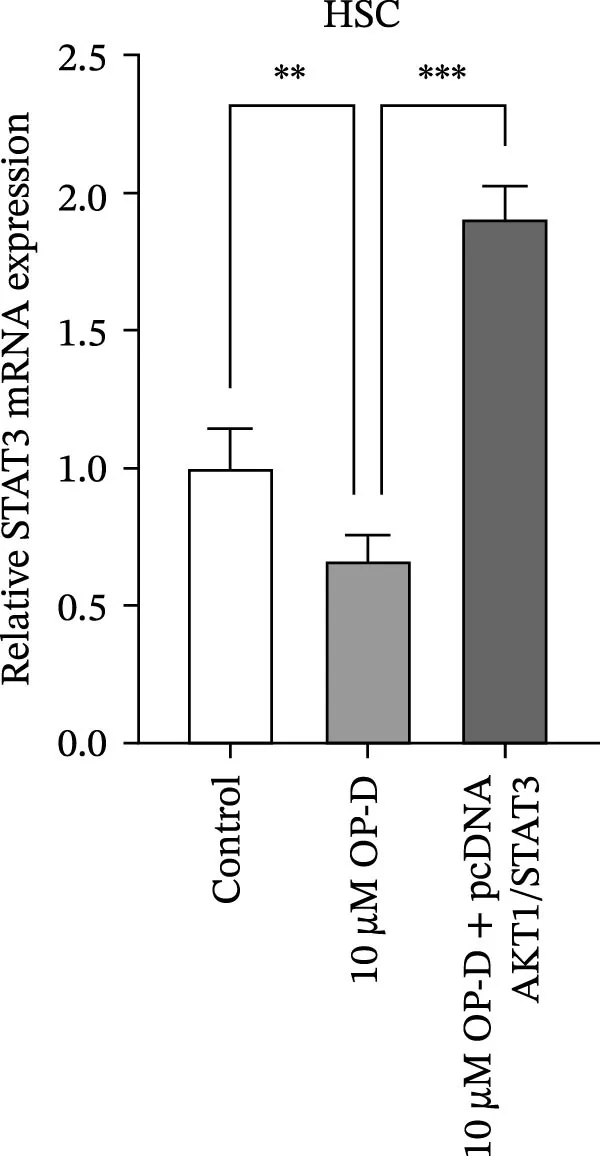
(B)

**Figure:**
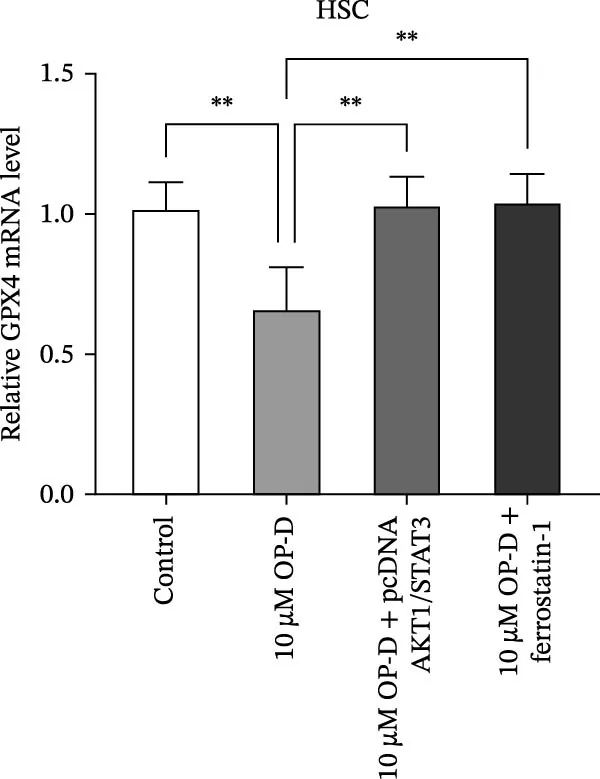
(C)

**Figure:**
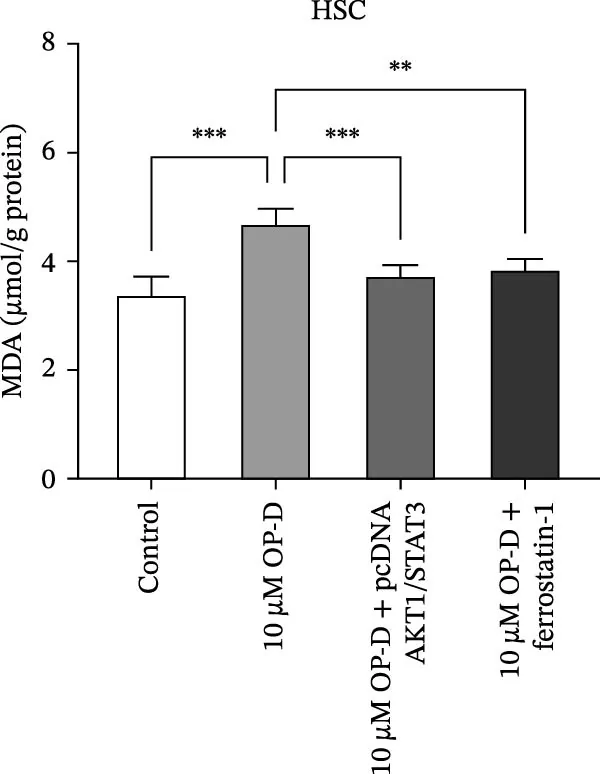
(D)

**Figure:**
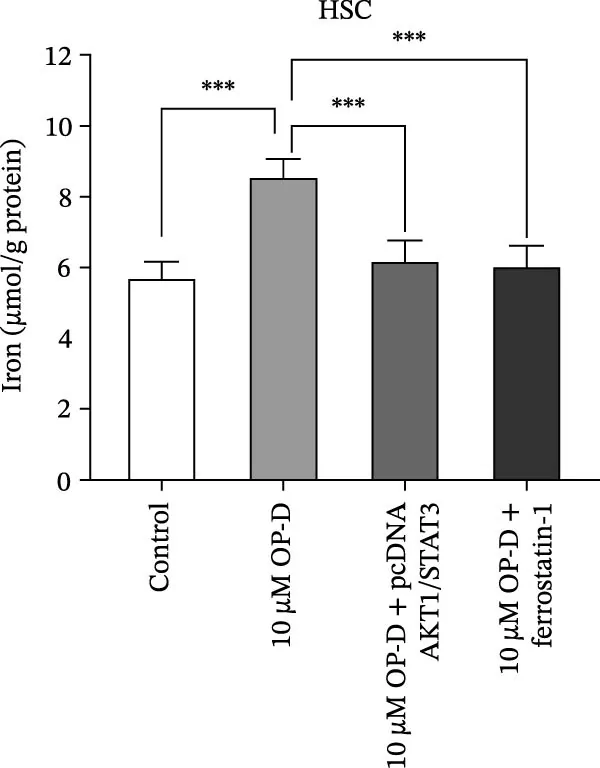
(E)

**Figure:**
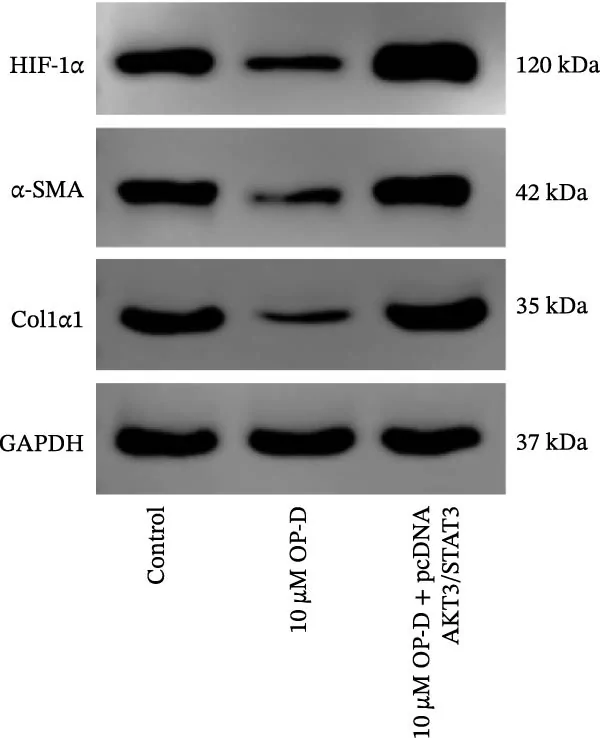
(F)

**Figure:**
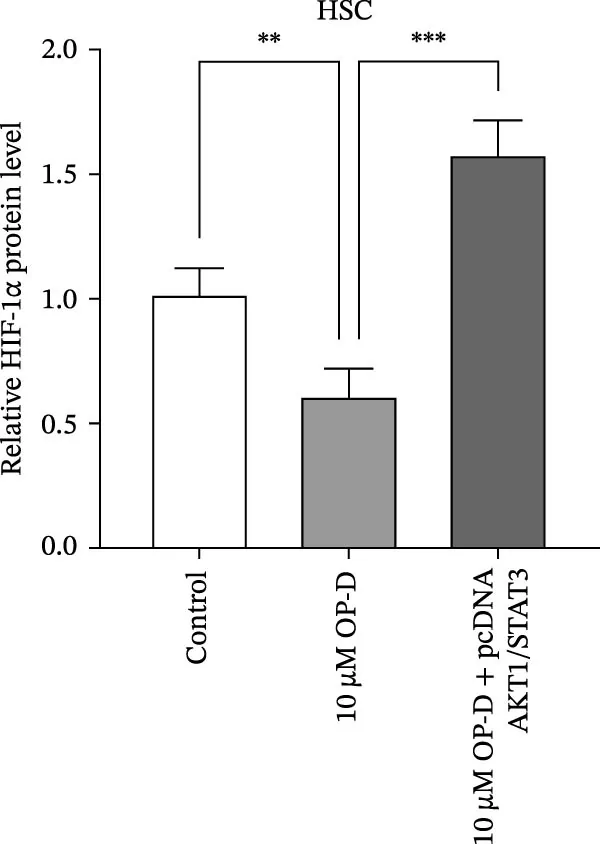
(G)

**Figure:**
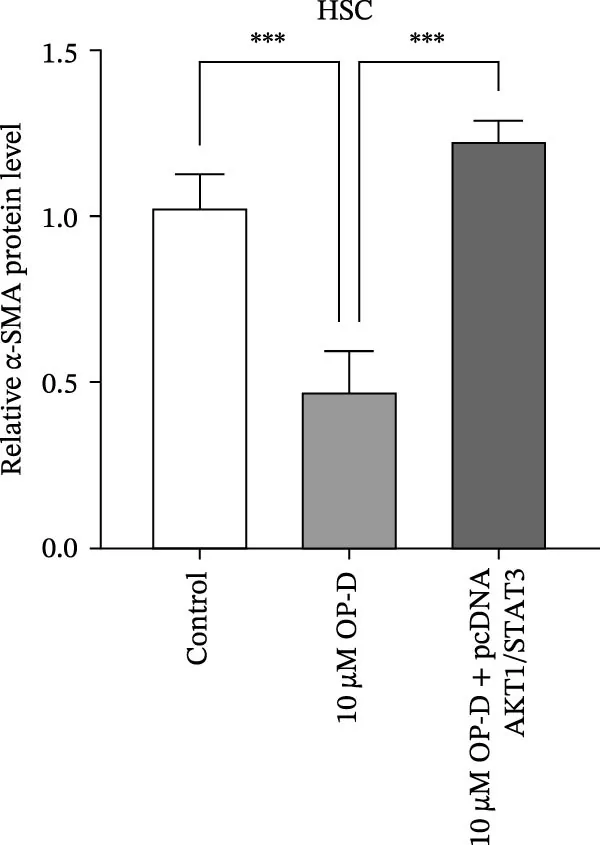
(H)

**Figure:**
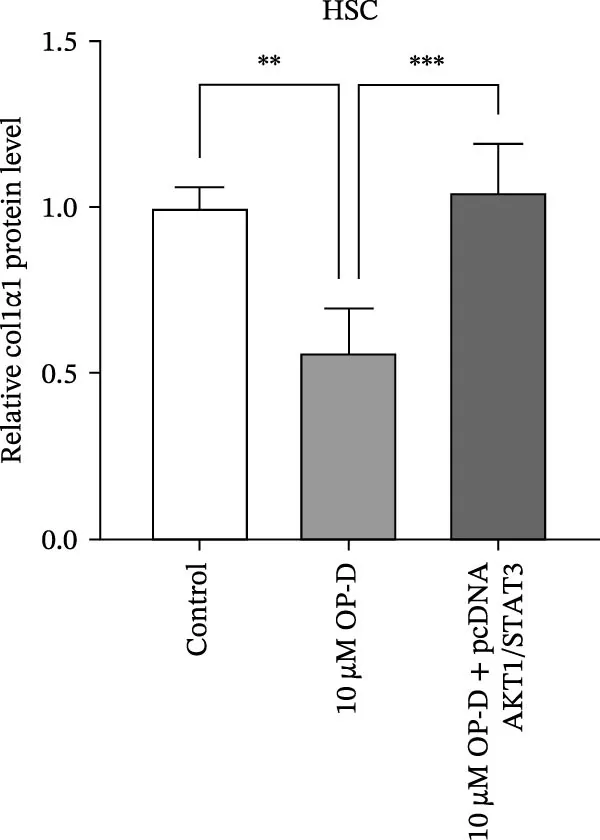
(I)

## 4. Discussion

In this study, we systematically investigated the therapeutic role of OP‐D in NASH and its underlying mechanisms using the HFD‐induced rat NASH model. Our results showed that OP‐D can alleviate HFD‐induced hyperlipidemia, inflammation, and fibrosis. Mechanistic studies suggest that OP‐D may induce ferroptosis by suppressing AKT1 and STAT3 in the HIF‐1 pathway.

Liver inflammation promotes lipid deposition in the liver, triggering liver inflammatory injury [22, 23]. In the treatment of NASH, improvement of hepatic inflammation is essential. It has been indicated that herbal medicine, including crude extract and bioactive compounds, possesses protective properties from inflammation, thus slowing down the NASH progression [11]. OP‐D can attenuate the release of proinflammatory cytokines stimulated by particulate matter of ≤2.5 µm in diameter in mouse pulmonary epithelial cells via suppressing the AMPK/NF‐κB pathway [24]. OP‐D can suppress renal inflammatory response induced by streptozotocin in diabetic rats [25]. OP‐D protects human umbilical vein endothelial cells from inflammatory injury via the CYP2J2/EETs‐PPARα pathway [26]. Here, we found that OP‐D ameliorated the inflammatory response in HFD‐fed rats and PA‐induced liver cells. These results were in line with those of Huang et al. [15] study. This study lacked a positive control compound for direct comparison, which limits the ability to benchmark the ferroptosis‐inducing efficacy of OP‐D against established inducers; however, the use of ferroptosis‐specific inhibitors provides mechanistic validation for the involvement of this pathway.

AKT1 was essential for dysregulation of inflammatory responses [27]. Gene silencing of AKT1 can alleviate NASH progression in vivo [28]. A potential causal relationship between elevated IL‐6/STAT3 activity and NASH susceptibility has been reported [29]. Notably, AKT1 and STAT3 were the targets of OP‐D. Therefore, OP‐D may ameliorate fibrosis in NASH via inhibition of AKT1 and STAT3.

HIF‐1α is a key transcription factor for cellular adaptive responses to hypoxia [30]. Under normoxia, HIF‐1α can be recognized and degraded via the ubiquitin protease pathway, so the level of HIF‐1α in normal cells is low. Under hypoxia, the ubiquitin degradation pathway is inhibited, and the increased expression of intracellular HIF‐1α activates the transcriptional expression of related target genes [31]. During liver fibrosis, hepatic stellate cell activation produces a large amount of extracellular matrix and overdeposits it, resulting in a pseudo‐hypoxic state of hepatocytes [32, 33]. Therefore, HIF‐1α expression was significantly elevated in several liver fibrosis models, and the magnitude of the elevation was positively correlated with the severity of liver fibrosis [33]. The reduction of HIF‐1α has been identified to induce hepatic stellate cell ferroptosis and attenuate the degree of fibrosis [34]. Here, we found the suppression of OP‐D on AKT1 and STAT3 caused a decrease in HIF‐1α and an increase in ferroptosis markers. Our results suggest that the AKT1/STAT3/HIF‐1α axis is a key mediating regulatory axis in OP‐D‐induced hepatic stellate cell ferroptosis and thus resistance to liver fibrosis.

In summary, our results indicate that OP‐D can alleviate liver fibrosis during NASH by regulating ferroptosis via the AKT1/STAT3/HIF‐1α axis. These results indicate that OP‐D may serve as a novel therapeutic agent for NASH patients in the future.

## Funding

This research received no specific grant from any funding agency in the public, commercial, or not‐for‐profit sectors.

## Ethics Statement

All animal experimental protocols were approved by and all experiments were performed according to the guideline of the Animal Ethics Committee of Dongguan TCM Hospital. This study was carried out in compliance with the ARRIVE (Animal Research Reporting In Vivo Experiment) guidelines.

## Consent

The authors have nothing to report.

## Conflicts of Interest

The authors declare no conflicts of interest.

## Data Availability

The data that support the findings of this study are available from the corresponding author upon reasonable request.
